# Development and Evaluation of Azelaic Acid-Loaded Microemulsion for Transfollicular Drug Delivery Through Guinea Pig Skin: A Mechanistic Study

**DOI:** 10.34172/apb.2020.028

**Published:** 2020-02-18

**Authors:** Anayatollah Salimi, Behzad Sharif Makhmal Zadeh, Salar Godazgari, Abbas Rahdar

**Affiliations:** ^1^Nanotechnology Research Center, Ahvaz Jundishapur University of Medical Sciences, Ahvaz, Iran.; ^2^Department of Pharmaceutics, Ahvaz Jundishapur University of Medical Sciences, Ahvaz, Iran.; ^3^Student Research Committee, Ahvaz Jundishapur University of Medical Sciences, Ahvaz, Iran.; ^4^Department of Physics, University of Zabol, Zabol, Iran.

**Keywords:** Azelaic acid, Microemulsion, Phase diagram, Follicular drug delivery, Acne vulgaris, Acne treatment

## Abstract

***Purpose:*** Azelaic acid is a natural keratolytic, comedolytic, and antibacterial drug that is used to treat acne. The topical application of azelaic acid is associated with problems such as irritation and low permeability. For dissolving, the problem is that microemulsion (ME) is used as a drug carrier. The aim of this study was to increase the azelaic acid affinity in the follicular pathway through ME.

***Methods:*** Azelaic acid-loaded MEs were prepared by the water titration method. The properties of the MEs included formulation stability, particle size, drug release profile, thermal behavior of MEs, the diffusion coefficient of the MEs and skin permeability in the non-hairy ear skin and hairy abdominal skin of guinea pig were studied in situ.

***Results:*** The MEs demonstrated a mean droplet size between 5 to 150 nm. In the higher ratios of surfactant/co-surfactant, a more extensive ME zone was found. All MEs increased the azelaic acid flux through both hairy and non-hairy skin compared with an aqueous solution of azelaic acid as a control. This effect of the ME was mainly dependent on the droplet diffusion coefficient and hydrodynamic radius. MEs with a higher diffusion coefficient demonstrated higher azelaic acid flux through hairy and non-hairy skin. Drug flux through both skins was affected by the surfactant/co-surfactant ratio in that the higher ratio increased the azelaic acid affinity into the follicular pathway.

***Conclusion:*** Finally, the ME with the highest droplet diffusion coefficient and the lowest surfactant/co-surfactant ratio was the best ME for azelaic acid delivery into the follicular pathway.

## Introduction

Acne vulgaris is a multifunctional disease of the pilosebaceous unit that may present at any age and it is associated with various clinical presentations such as comedones, papules, pustules and nodules. Acne has four main pathogenetic mechanisms including increased sebum production, hyper-keratinization, the colonization of *Propionibacterium acne* in the follicles and inflammation.^1^ Acne treatment is mainly done in three different ways including topical therapy, systemic treatment and other treatments like dermabrasion, xenografts, autografts, and chemical peeling. Topical treatment is the main choice for mild and moderate acne.^2^ Most of the conventional topical formulation usually demonstrates a high incidence of side effects that reduce patient compliance and thus the efficacy of the therapy. Azelaic acid is an effective compound for the topical treatment of mild to moderate acne vulgaris.^3^ The therapeutic Formula 20% azelaic acid cream formulation has been proven in the treatment of acne (comedone, pustular, nodular) but its therapeutic effects may not be seen for up to 4 weeks after use. The patient should continue therapy for more than 6 months.^4^ After the topical application of 1 g of 20% azelaic acid cream, a percutaneous absorption of about 3% and plasma concentration of 0.038 µg/mL were estimated.^5^ Therefore low dermal absorption diminishes its therapeutic effectiveness. The site of action for azelaic acid is a pilosebaceous unit and its accumulation in this area can improve acne treatment. The azelaic acid concentration in the hair follicles is affected by the nanocarriers.

Microemulsion (ME) is an isotropic and thermodynamically stable dispersion including the oil phase, surfactant, cosurfactant, and aqueous phase. MEs have been used as a carrier for transdermal drug delivery because of their ability to increase drug solubility and induce a high concentration gradient toward the skin. MEs present several advantages consisting of ease of preparation, drug solubilization capacity, and the permeation enhancer effect by changing the stratum corneum barrier property. Some of the materials in ME may act as enhancers by disrupting the structure of stratum corneum. This feature is more related to the properties of some of the components, such as oils and surfactants, rather than the main structure of the ME itself. MEs have a high potential for favoring the solubility of hydrophilic and lipophilic drugs and it may increase the thermodynamic activity of the drug in the skin and thus increase its skin penetration.^6-8^

Despite the great benefits of dermal and transdermal drug delivery, only a limited number of drugs are available today as topical formulations. The most crucial cause is the skin’s barrier, which is known as the most impervious to the epithelial membrane of the human body to external compounds. Therefore, it is important to provide adequate penetration of the drug into the skin without inducing irreversible changes in the function of the skin barrier.^9^

Conventional topical formulations of azelaic acid demonstrated limitations such as stinging, irritation, burning, itching and purpuritis. Carrier-based drug delivery systems can reduce these side effects by gradual drug release and by dispersing the drug through the different layers of skin that reduce the local concentration.^10^ Therefore, preparation and optimization of ME for follicular delivery of azelaic acid for increasing the efficacy and decreasing the side effects was the main aim of this study.

## Materials and Methods

### Materials

Azelaic acid was provided from the Sepidaj company (Tehran, Iran), Caprylocaproylmacrogoglycerides (Labrasol), Capryol 90 (Propylene glycol monocaprylate) and Diethylene glycol monoethyl ether (Transcutol P) were gifts presented by (Gattefosse, Saint-Priest, France). Oleic acid, tween 80 and propylene glycol were purchased from Merck (Germany). Minitab17 software was used for experimental design and the other statistical evaluations. Ternary phase diagrams were obtained by Sigma plot 14.

### Animal studies

Female adult guinea pigs ranging in weight from 600-800 g at the age of 20-22 weeks were purchased from Razi Institute, Karaj, Iran. Animals were given a standard diet and water and then were used based on principles for the care and use of laboratory animals. The guidelines followed were those laid down by the National Academy of Sciences and published by the National Institutes of Health (U.S. Department of Health & human services, the office of laboratory animal welfare).

### The solubility of azelaic acid

The solubility of azelaic acid in oleic acid and oleic acid +Transcutol P (10:1) and acid oleic+ surfactants (Tween 80, Labrasol,) and co-surfactant (Capryol 90) was calculated by dissolving an excess amount of azelaic acid in 5 mL of acid oleic or acid oleic and surfactant, and cosurfactant mixture. The samples were mechanically mixed for 48 h at 25 ± 0.5°C. After equilibration, the clear supernatants were filtered through a polytetrafluoroethylene membrane filter (0.45 μm), and the filtrates were analyzed by UV spectrophotometer at 210 nm.^11^

### ME Preparation based on phase diagram construction

A pseudoternary phase diagram was prepared using the water titration method to distinguish between the zones of ME formation.

Based on the 4:1 and 6:1 weight ratios of (Labrasol-Tween 80/Capryol 90), two-phase diagrams were constructed. The oil phase (oleic acid + Transcutol-P) ratio (10:1) to the mixture of surfactant and cosurfactant (Tween80-labrasol/Capryol 90) was designed as 1:9, 2:8, 3:7, 4:6, 5:5, 6:4, 7:3, 8:2, and 9:1. The mixture of the oil phase, surfactant, and cosurfactant was titrated drop-by-drop using deionized water under agitation. After equilibration, the transparent and homogenous samples were considered to be a ME area in the phase diagram.^12,13^

The properties of the MEs may be affected by surfactant/co-surfactant ratio (S/C), water percent (%w) and oil percent (% oil). A full factorial design was used concerning the 3 variables at 2 levels in this study and 8 MEs with 5 and 10% oil, 20-30% water, 4:1, 6:1 S/C ratio and 1% azelaic acid were prepared (Table 1).

**Table 1:** Composition of selected MEs of Azelaic Acid

**Batch No.**	**Factorial**	**S/C**	**% Oil**	**%(S/C)**	**% Water**
ME-1	+++	6:1	10	60	30
ME-2	++-	6:1	10	70	20
ME-3	+-+	6:1	5	65	30
ME-4	+--	6:1	5	75	20
ME-5	---	4:1	5	75	20
ME-6	--+	4:1	5	65	30
ME-7	-+-	4:1	10	70	20
ME-8	-++	4:1	10	60	30

The oil phase including 1% azelaic acid was mixed with S+C mixture and then drop by drop double distilled water was added to the mixture stirring at an ambient temperature until a uniform mixture was obtained.^13^

### Droplet size measurement

Particle size was measured by SCATTER SCOPE 1 QUIDIX (South Korea) based on photon correlation spectroscopy at 25°C with a wide measurable size range (1-7000 nm).

### Dynamic light scattering (DLS)

DLS is a tool to characterize dynamic parameters of MEs such as diffusion coefficient and particle size. The scattered light intensity is a fluctuating and time-dependent phenomenon that depends on the size, Brownian motion and diffusive behavior of particles in solution and viscosity of continuous phase. These fluctuations are defined by the normalized autocorrelation function, g^1^ (τ), of the scattered electrical field, forgiven delay time, τ, which provides information about the structure and dynamics of the scattered particles.^14-16^

(1) $$g^{1}\left(q.\tau \right)=\frac{E\left(q.t\right)E^{*}\left(q.t+\tau \right)}{I\left(q.t\right)}$$

in this equation, E* is the complex conjugate of E. Experimentally, the intensity autocorrelation function, g^2^(q, τ), is calculated as following^17-19^:

(2) $$g^{2}\left(q.\tau \right)=\frac{E\left(q.t\right)E^{*}\left(q.t\right)E\left(q.t+\tau \right)E^{*}\left(q.t+\tau \right)}{I^{2}\left(q.t\right)}$$

Using the Siegret relationship, the normalized autocorrelation function, g^2^(q,τ), is changed to the autocorrelation function of the scattered electrical field, g1 (q, τ).

(3) $$g^{2}\left(q.\tau \right)=1+{\left|A\exp\left(-\Gamma \tau \right)\right|}^{2}$$

Here, *A* is an instrumental constant. For a ME, the function of g1(q, τ) is represented by a single exponential decay curve.^16-18^

(4) $$g^{2}\left(q.\tau \right)=1+\left|A\exp\left(-\Gamma \tau \right)\right|$$

The decay rate, *Γ*, is converted to the diffusion coefficient.

Finally, the diffusion coefficient of nanoparticles can be characterized as the hydrodynamic size according to the Stokes-Einstein relation^19^:

(5) $$r_{h}=\frac{K_{B}T}{6\eta \pi D}$$

in this equation: *K* is Boltzmann’s constant, T is the temperature in K, and η is the viscosity of the solvent.

### Viscosity determination

The rheology behavior of MEs was evaluated by Brookfield viscometer (DV-II + Pro Brookfield, USA) at 25 ± 1°C and spindle 34, at 75 rpm. A 10 mL volume sample was used for viscosity measurements.^20^

### pH measurement

The apparent pH values of the MEs were measured at 25ºC using a calibrated glass electrode (Mettler Toledo seven easy, Switzerland).

### Physical stability studies

Based on ICH guidelines, MEs were placed in different temperatures (4°C, 25°C, 37°C and 75% ± 5% RH for 6 months) and then visually inspected for phase separation, precipitation. Furthermore, in a centrifugation test at 15 000 rpm for 30 min at 25 ± 1°C (MPV-350R, Poland), the samples were visually inspected.^13^

### Thermal behavior evaluation by differential scanning calorimetry (DSC)

The thermograms were provided by a Mettler Toledo DSC star^®^ system. A defined amount of each ME sample was weighted and sealed into the aluminum pan and exposed to temperature between+30°C to −50°C (scan rate: 10°C/min) against an empty hermetically sealed pan. Based on thermograms, transition phase temperatures and enthalpies (ΔH) were calculated.^9^

### Drug release experiment

Specially designed static Franz diffusion cells (area 3.4618 cm^2^) equipped with a cellulose membrane (3000-4000 KD cut-off) were applied to calculate the release rate of azelaic acid through different MEs.

Five grams azelaic acid loaded ME was accurately weighed and placed on the membrane. Phosphate buffer solution (PBS) pH 7 was used as a receptor medium. The receptor fluid was stirred at 200 rpm by magnetic bars at definite time intervals (0.5, 1, 2, 3, 4, 5, 6, 7, 8, and 24 h), 2 mL sample was removed from receptor compartment and the released drug determined at 210 nm. The cumulative percentage of the released drug was plotted versus time, and release data fitted on zero, first and Higuchi kinetic models. T model was chosen based on the maximum coefficient correlation (r^2^).^21^

### Permeability experiments

To investigate the azelaic acid ME permeation amount through the follicular and epidermal pathways, the hairy abdominal skin and non-hairy pig ear skin regions were used as epidermal and follicular pathways.^22^

Vertical diffusion cells (with an effective diffusion area of approximately 3.4618-0.3846 cm^2^) were used for *in vitro* permeation through hairy and non-hairy guinea pigs. The receptor compartments were filled with 28 and 10 mL PBS (PBS, pH 7) respectively. The hairy abdominal skin and non-hairy pig ear skin samples, hydrated before use, were mounted between the donor and receptor compartments of the cells with the stratum corneum facing the donor medium. The donor phases were filled with azelaic acid-loaded MEs (5 g and 0.5 g). The diffusion cells were placed in a water bath equipped with a magnet stirrer (37 ± 0.5°C) and the receptor phase was stirred at 200 rpm. At each interval time (0.5, 1, 2, 3, 4, 5, 6, 7, 8, 24 and 48 h), 2 mL and 0.5 mL samples were withdrawn from the receptor mediums and replaced with fresh PBS. A UV spectrophotometer was utilized to detect the permeated amount of azelaic acid in the samples derived at 210 nm. The aqueous solution of azelaic acid with the same concentration as the MEs was used as a control. The results were plotted as a cumulative permeated drug percentage versus time. Based on these plots, the apparent permeability coefficient (Eq. 6) and steady-state permeation flux (J_ss_) (Eq. 7) were calculated.

(6) $$P_{app}=\frac{dQ}{dt}\times \frac{1}{A.C_{0}}$$

(7) $$J_{SS}=C_{0}\times P_{app}$$

*dQ/dt* is the steady-state appearance rate on the acceptor side of the skin. *A* is the area of the skin and C_0_ is the initial concentration of the drug in the donor phase.

### Data analysis

The steady-state permeation rate (J_ss_) of azelaic acid through the unit skin area was obtained from the linear portion of the slope of the permeation curve. One-way analysis of variance (ANOVA) was used to see any significant differences and *P* < 0.05 was considered to be significant with 95% confidence intervals.

## Results and Discussion

### Azelaic acid solubility

The solubility of azelaic acid is tabulated in Table 2.

**Table 2:** The solubility of Azelaic acid in different oils, surfactant, and co-surfactants (mean±SD, n=3)

**Phase type**	**Excipient**	**Solubility (mg/mL)**
Oil	Oleic acid	0.182±0.07
	Transcutol-P	3.02±0.22
Surfactant	Tween 80	0.313±0.14
	labrasol	4.09±0.2
Co-surfactant	Capriol 90	0.19±0.03

### Phase behavior studies

**Figure 1:**
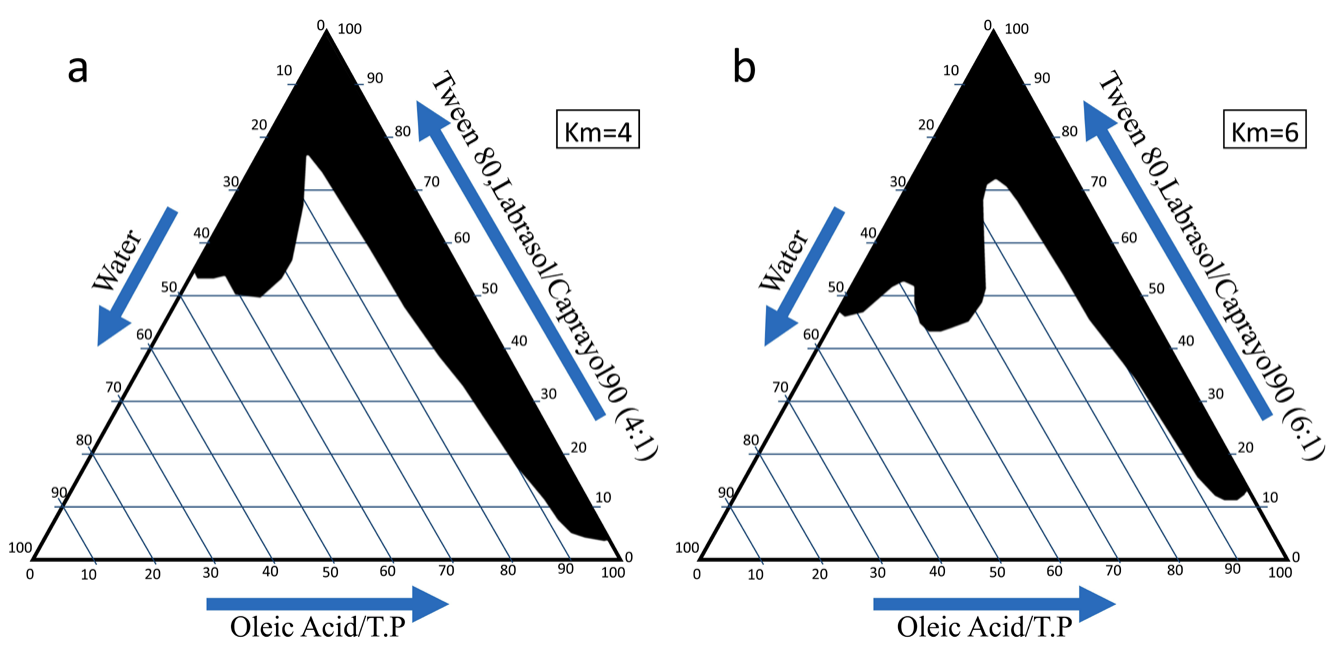
The pseudo-ternary phase diagrams of the oil-surfactant/cosurfactant mixture–water system at the 4:1 and6:1weight ratio of Labrasol -Tween 80/ Capryol 90 at ambient temperature, dark area show microemulsions zone.

For the determination of the ME zone based on the different S/C ratios two phase, diagrams were drawn (Figure 1). The weight ratio of S/C is an important parameter affecting the phase behaviors of the ME. The increase in ME region was provided by a higher concentration of surfactant. The phase diagrams clearly indicated that the ME existence region increased with an increase in the weight ratio of S/C and this led to the incorporation of more water in the ME structure (Km=4-6).

### Properties of AZ MEs

#### Viscosity and particle size of MEs

The viscosity, mean droplet size, polydispersity index and the pH of the azelaic acid MEs has been presented in Table 3.

**Table 3:** pH, mean droplet size, polydispersity index and Viscosity of Azelaic Acid MEs (mean±SD, n=3)

**Formulation**	**pH**	**Mean droplet size (nm)**	**Polydispersity index**	**Viscosity(cps)**
ME-1	4.69±0.08	48.05±0.75	0.34±0.02	78±0.03
ME-2	5.05±0.07	94.05±0.65	0.351±0.011	83±1.4
ME-3	4.79±0.04	65.85±3.8	0.363±0.009	72.5±1.45
ME-4	5.15±0.06	151±1.1	0.348±0.011	83±1.7
ME-5	4.64±0.03	103±2.3	0.341±0.017	75±1.84
ME-6	4.88±0.04	89.3±5.2	0.347±0.013	74.4±0.9
ME-7	4.81±0.03	121.5±1.66	0.348±0.001	77.6±1.75
ME-8	4.59±0.04	107.65±2.7	0.337±0.006	76.5±1.62

The mean droplet size of the MEs was from 48 to 151 nm (Table 2). The ME-1 had the lowest particle size (48.05 ± 0.75 nm) with a narrow size distribution (Table 2).

The statistical analysis showed a significant (*P* <0.05) and indirect correlation between the mean droplet size and the percentage of water (%W). On the other hand, a significant and indirect correlation was found between the viscosity of MEs with a %W and S/C ratio. The viscosity values proposed Newtonian behavior in all of the MEs. The MEs had a pH value (5.305±0.09) that is compatible with the skin (Table 3).

### Azelaic acid release profiles

**Figure 2:**
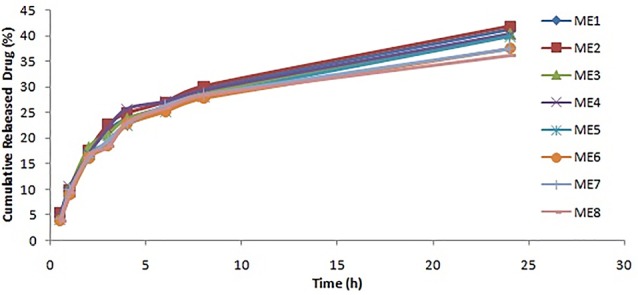
In vitro release profile of MEs of Azelaic acid (Mean ± SD, n=5).

The percentage of the released drug is a critical characteristic of the formulation which plays an important role in therapeutic effectiveness. Figure 2 shows the release profiles of the azelaic acid-loaded MEs. The drug release profile showed that 36%-42% of the drug was released during the 24 h of the experiment. The azelaic acid release study thus suggests a controlled release profile that is useful for producing a sensible drug depot into the skin. Higuchi was the best fitting model for the release data. This means that the diffusion controls the drug release rate. Previously, a zero-order model was reported for the azelaic acid release through ethosomes.^10^ In comparison between the MEs and ethosome, it seems that the solubility of azelaic acid in the ethosomes was not complete and that the solubility was a rate-controlling step. The solubility of the azelaic acid in MEs was complete and so diffusion was the rate-limiting step.

### DSC thermograms of azelaic acid loaded MEs

Table 4 shows transition temperatures and transition enthalpies of azelaic acid loaded MEs. DSC study was used for water behavior in MEs and distinction between bulk (free) and bound (interfacial) water.^23^

**Table 4:** Results of the Diffusion and Hydrodynamic size, enthalpy (ΔH) and transition temperatures (Tm) of drug-loaded MEs (Mean±SD, n=3)

**Formulation No.**	**Diffusion (m** ^ 2 ^ **/s)**	**Hydrodynamic radius (nm)**	**Temperature (°C)**	**ΔH (Mj/mg)**
ME-1	7.32308E-12	32.6	−34.9±0.1	69.78±0.01
ME-2	2.52093E-12	94.7	−39.5±0.5	52.8±0.36
ME-3	2.75355E-12	86.7	−35.5±0.5	160 ±0.35
ME-4	1.57061E-12	152	−32.9±0.1	141.6±0.4
ME-5	4.39655E-12	54.3	−24.75±0.25	179.8±0. 5
ME-6	3.07645E-12	77.6	−27.75±0.25	132.5±0.9
ME-7	1.80858E-12	132	−28.8±0.2	127.9±1.2
ME-8	2.45357E-12	97.3	−29.65±0.45	150±1. 1

In cooling curves of the ME formulations, bulk water (free water) and bound water were obtained at 0°C and −24.75 to −39.5°C, respectively.

According to ANOVA results, a significant correlation (*P* ˂ 0.05) was found between the bound melting transition temperature (Tm_2_) and independent variables, in this manner that higher Tm_2_ provided by the lower amount of oil and S/C ratio. The same correlation was found between the oil percent and ΔH_2_. Our findings are in agreement with the previous reports by Podlogar et al.^24^ They reported the transition temperatures of the free water and bound water around −8 to 0°C and −17 to −26°C, respectively.

The thermal behavior of water is an applicable tool for evaluation of the microstructure of MEs.^23^ An exothermic peak at low temperatures (between -32.9ºC to -39.5 in MEA-1 to MEA-4 with higher surfactant) has been reported as water that strongly interacts with the surfactants.^9,13^

### MEs physical stability

The ME formulations were found to be physically stable for a period of 6 months with no phase separation, flocculation or coalescence during storage at different temperature conditions and during centrifugation.

### DLS results

**Figure 3:**
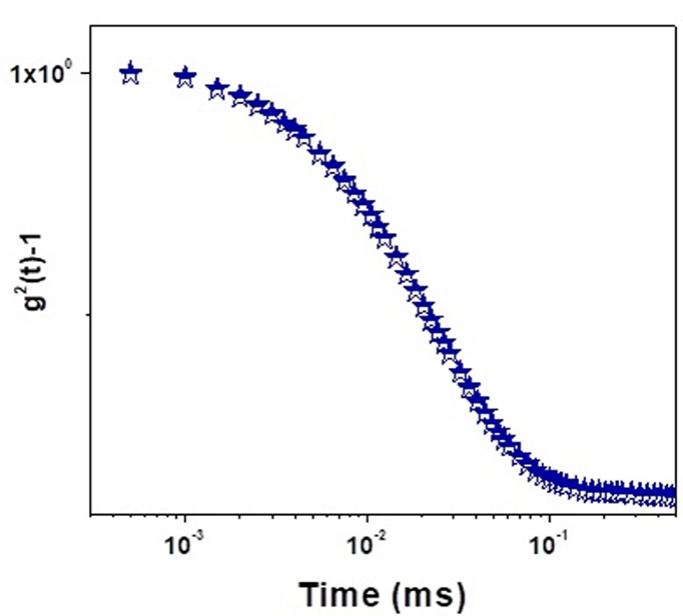
The autocorrelation function of drug-loaded MEs as a function of relaxation time at RT.

The autocorrelation function of the drug-loaded ME as a function of relaxation time at RT for sample No.5 as an example has been presented in Figure 3.

The results related to the diffusion and hydrodynamic size of the azelaic acid-loaded MEs as a function of relaxation time at RT according to the theory of DLS have been shown in Table 4. A dramatically inverse correlation was found between the droplet diffusion coefficients and hydrodynamic radius. The highest diffusion coefficient (ME 1) provided the lowest hydrodynamic radius. The diffusion coefficient of the ME droplets may be affected by different parameters. Matsaridou et al reported that high viscosity restricted droplet mobility and diffusion coefficient through the skin.^25^ The lack of significant difference between the viscosities of the MEs in the present study showed that viscosity did not significantly affect the droplet diffusion coefficient. Bedwell and Gulari reported the correlation between the diffusion coefficient and the disperse phase concentration.^26^ Similar results were found in the present study. A significant correlation was found between the water and oil percentage with droplet diffusion coefficients in the same manner. This means that water and oil can play a role in the disperse phase and most of MEs demonstrated bicontinuous structures.

### In-vitro permeation studies through guinea pig skin

The hairy abdominal skin and non-hairy guinea pig ear skin regions were used as a model for the epidermal and follicular pathways.^22^ Therefore in this study, permeation studies were done on both hairy and non-hairy guinea pig skin.

The permeability parameters of the various azelaic acid- loaded MEs through hairy abdominal skin (except MEA-1) were less than non-hairy ear skin pig (Table 5). This is in accordance with the hydrophilic nature of azelaic acid. It seems that the MEs significantly increased the azelaic acid solubility into the epidermal route. On the other hand, the permeability parameters of azelaic acid in the control through the hairy skin were the same as the non-hairy skin. In both skins, less than permeable parameters were provided by the azelaic acid- loaded MEs (except ME 8).

**Table 5:** *In vitro* permeability parameters of azelaic acid-loaded MEs through hairy abdominal and non-hairy ear skin guinea pig (Mean±SD, n=5)

**Formulation**	**Hairy Abdominal skin**		**Non-hairy ear skin**	
	**J**_ss_ **(mg/cm** ^ 2 ^ **.h)**	**P (mg/cm** ^ 2 ^ **)**	**J**_ss_ **(mg/cm** ^ 2 ^ **.h)**	**P (mg/cm** ^ 2 ^ **)**
Control	0.481±0.018	0.0069±0.0003	0.4968±0.018	0.006±0.0002
ME-1	1.43±0.17	0.0143±0.0017	0.92±0.04	0.009±0.0004
ME-2	0.896±0.071	0.0089±0.0007	2.12±0.29	0.021±0.002
ME-3	0.734±0.06	0.0073±0.0006	1.38±0.004	0.0138±0.00004
ME-4	0.826±0.07	0.0082±0.0007	1.32±0.78	0.0132±0.007
ME-5	1.076±0.30	0.0707±0.003	1.47±0.02	0.0147±0.0002
ME-6	0.847±0.26	0.0084±0.002	1.184±0.04	0.0118±0.0004
ME-7	0.611±0.29	0.0061±0.002	1.6±0.26	0.0160±0.002
ME-8	0.232±0.046	0.0023±0.0004	0.56±0.17	0.0056±0.001

Among the MEs, ME-1 showed the highest skin permeability. The J_ss_ of the azelaic acid loaded- ME1 (1.43 mg cm^-2^ h^-1^) was 2.88 folds higher than the control through the abdominal skin of a guinea pig. In this study, the other parameters like Q_8h_, Q_48h_ (the amount of drug permeated per surface area after 8 and 48 h) and Ratio_8h_, Ratio _48h_ (the Q_8_ and Q_48_ produced by MEs compared with control) have been indicated in Table 6. MEs 1 had the highest Q_8h_ and Q_48h_ in both pathways.

**Table 6:** Amount of drug permeated per unit area in MEs compared with the aqueous saturated solution (control) through hairy and non-hairy pig skin (Mean±SD, n = 5)

**Formulation No.**	**Hairy Abdominal skin**		**Non-hairy ear skin**			
	**Q**_8h_ **(mg/cm** ^ 2 ^ **)**	**Q**_48h_ **(mg/cm** ^ 2 ^ **)**	**Q**_8h_ **(mg/cm** ^ 2 ^ **)**	**Q**_48h_ **(mg/cm** ^ 2 ^ **)**	**Ratio**_8h_	**Ratio**_48h_
Control	1.228±0.04	2.42±0.062	1.228±0.04	2.421±0.062	-	-
ME-1	3.04±0.42	5.05±0.23	9.35±0.23	15.4±0.78	3.07±0.03	3.04±0.01
ME-2	2.63±0.57	4.32±0.1	7.98±0.24	13.45±0.39	2.8±0.2	3.1±0.16
ME-3	2.36±0.06	4.2±0.02	7.03±0.05	12.21±0.17	2.9±0.1001	2.91±0.06
ME-4	2.29±0.01	3.75±0.05	6.42±0.05	10.75±0.25	2.8±0.005	2.86±0.02
ME-5	2.15±0.032	3.67±0.04	6.03±0.14	10.23±0.06	2.80±0.02	2.78±0.01
ME-6	2.35±0.04	2.99±0.04	6.73±0.1	11.9±0.21	2.86±0.09	2.98±0.01
ME-7	1.9±0.92	3.05±0.47	7.63±0.25	12.98±0.22	4.49±0.04	4.79±0.2
ME-8	1.38±0.014	2.26±0.03	8.93±0.06	14.92±0.55	6.44±0.01	6.48±0.15

ANOVA showed that the correlation between Q_8h_, Q_48h_ in the non-hairy ear skin with the independent variables (%W, S/C ratio, and %Oil) was significant (*P* < 0.05). The higher Q_8h_ and Q_48h_ were produced by a higher amount of S/C ratio, oil, and water percentage. The correlation between Q_8h_ with the S/C ratio in the hairy abdominal skin was significant (*P* < 0.05). The results suggested that in the hairy pathway, the effect of the surfactant concentration on drug permeation is only important and the effect of oil and water is negligible. Our findings are in agreement with the previous reports by Bhatia et al^27^ and Ma et al.^28^ In a study carried out by Peira et al, loaded azelaic acid in ME was used for topical application on pig skin.^29^ They showed that the azelaic acid flux provided by ME was higher than the azelaic acid solution. However, they did not present any data about the azelaic acid accumulation in the follicles. They also did not study the effect of the ME properties such as hydrodynamic diameter and droplet diffusion on the azelaic acid permeation through pig skin. In the present study, which is mechanistic, azelaic acid accumulation in the follicles was proved by ME.

It has been demonstrated that the penetration depth of the particles through the skin can be influenced by their size.^30^ For this purpose, the correlation between the ME droplet diffusion coefficient and the droplet radius with J_ss_ through hairy and non-hairy skin was studied. Based on the paired- t-test, the correlation between droplet diffusion coefficients and J_ss_ in hairy (*P* = 0.002) and non-hairy (*P* = 0.02) was both direct and significant. The values for the correlation between droplet radius and J_ss_ in the hairy and non-hairy skin were 0.00001 and 0.0009 respectively. It seems that the azelaic acid penetration through the skin is controlled by the droplet diffusion coefficient and the droplet radius, with the effect of the droplet radius being more important than the droplet diffusion coefficient. In comparison between hairy and non-hairy skin, the data demonstrated that the effect of the diffusion coefficient and droplet radius on the hairy skin is more than the non-hairy skin. Therefore, particle size is a critical criterion for azelaic acid penetration through the epidermis and follicular routes. The highest droplet diffusion belongs to formulation 1, which provided the highest J_ss_ in hairy skin. In non-hairy skin, the highest J_ss_ was provided by ME 2. The difference between ME-1 and 2 is only the amount of surfactant. ME 2 has the higher amount of surfactant compared with ME 1 provided that the highest J_ss_ was on the non-hairy skin. This means that ME 2 altered the epidermis barrier. The effect of the surfactant on the barrier property of the epidermis was more than on the follicular route. The follicular route is the main acting site of azelaic acid in acne. Therefore more azelaic acid penetration in the hairy route is the goal of this study that was provided by ME-1 with the highest droplet diffusion coefficient.

## Conclusion

Azelaic acid is an anti-acne agent with poor permeation through the skin that diminishes its therapeutic effectiveness and increases the skin irritant potency. In this study, azelaic acid- loaded MEs were used to get a higher azelaic acid concentration in the follicles as the main action site in acne treatment. The MEs increased the azelaic acid penetration into the epidermal and follicular pathways compared with the aqueous azelaic acid solution. Different MEs provided different J_ss_, Q_8h_, and Q_48h_ enhancement ratios in the two pathways. In both pathways, MEs with higher droplet diffusion coefficients and lower hydrodynamic radius cased higher azelaic acid penetration compared with the control. MEs, with the higher amount of surfactant increasing the azelaic acid’s affinity for penetration through the epidermal pathway. Based on the fact that the main site for azelaic acid action in acne is the follicular pathway, the ME-1 with a low amount of surfactant, low hydrodynamic radius and high droplet diffusion coefficient was the best ME that increased the azelaic acid affinity to the follicular route.

## Ethical Issues

Approval for the studies was given by the Ethical Committee of the Ahvaz Jundishapur University of Medical Sciences.

## Conflict of Interest

There is no declaration of interest.

## Acknowledgments

This study was carried out under financial support provided by Ahvaz Jundishapur University of Medical Sciences (grant No 1068). We thank the vice chancellor for research and technology of AJUMS. The authors are thankful for Iranian Representation for Gattefosse Pharmaceuticals (Faratin Company).
